# Effects of Metabolic Dysfunction–Associated Steatohepatitis in Alertness, Associative Learning, and Astrocyte Density

**DOI:** 10.1002/brb3.70222

**Published:** 2024-12-31

**Authors:** Sara G. Higarza, Marina De Antón‐Cosío, Candela Zorzo, Jorge L. Arias, Natalia Arias

**Affiliations:** ^1^ INEUROPA, Instituto de Neurociencias del Principado de Asturias Oviedo Spain; ^2^ Neuroscience Laboratory, Department of Psychology University of Oviedo Oviedo Spain; ^3^ ISPA, Instituto de Investigación Sanitaria del Principado de Asturias Oviedo Spain; ^4^ BRABE Group, Department of Psychology, Faculty of Life and Natural Sciences University of Nebrija Madrid Spain

**Keywords:** astrocytes, metabolic dysfunction–associated steatohepatitis, passive avoidance, prepulse inhibition

## Abstract

**Purpose:**

Metabolic dysfunction–associated steatohepatitis (MASH) is a prevalent disease caused by high fat and high cholesterol intake, which leads to systemic deterioration. The aim of this research is to conduct a psychobiological exploration of MASH in adult male rats.

**Methods:**

Subjects who were administered a high‐fat and high‐cholesterol diet for 14 weeks. Then, we assessed the acoustic startle response and alertness through the prepulse inhibition paradigm as well as the associative learning by the use of the passive avoidance test. Also, we explored the astrocyte density in the prefrontal cortex and hippocampus.

**Results:**

Our results showed that, whereas the MASH group did not display an impaired associative learning, a lower exploration rate was found in this group. Moreover, a reduced prepulse inhibition was found in these subjects in the case of the weaker and closer‐to‐the‐stimulus prepulse, which indicates a mild alteration in this process. No differences were found in astrocyte density in the MASH group in comparison with controls.

**Conclusion:**

MASH seems to be linked with cognitive dysfunction. Further research is needed to elucidate the pathway involved in this disease and its underlying mechanism, as well as the potential implication in human health.

## Graphical abstract

Metabolic dysfunction‐associated steatohepatitis (MASH) is associated with a mild alteration in the prepulse inhibition test, indicating impaired alertness. Additionally, MASH resulted in reduced exploration in the passive avoidance test, without affecting associative learning. No significant differences in astrocyte density were observed in the MASH group compared to controls.

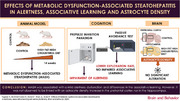

## 1 Introduction

Metabolic dysfunction–associated steatotic liver disease (MASLD) has emerged as the most prevalent chronic liver disease worldwide, being defined as the accumulation of excess triglycerides in the liver in the presence of at least one cardiometabolic risk factor (European Association for the Study of the Liver [EASL] et al. 2024; Z. Younossi et al. 2017). MASLD is considered the new term that replaces the concept of nonalcoholic fatty liver disease (NAFLD) and is part of a broader definition of steatotic liver disease. Thus, this condition is associated with an increased risk of cardiovascular events, chronic kidney disease, both hepatic and extrahepatic malignancies, and liver‐related outcomes such as liver failure and hepatocellular carcinoma (EASL et al. 2024).

The course of MASLD is considered a chronic metabolic disorder associated with multiple conditions such as dyslipidemia, central obesity, cardiovascular disease, hypertension, hyperglycemia, insulin resistance, Type 2 diabetes, chronic kidney disease, and genetic predisposition—such as genes involved in cellular lipid metabolism in the liver (Byrne and Targher 2015; Cobbina and Akhlaghi 2017; Croci et al. 2019; Juanola et al. 2021; Mikolasevic et al. 2016; Pouwels et al. 2022). MASLD prevalence ranges between 15% and 35% (Croci et al. 2019; Ipsen, Lykkesfeldt, and Tveden‐Nyborg 2018; Le et al. 2017; Paternostro y Trauner 2022) while metabolic dysfunction–associated steatohepatitis (MASH) prevalence ranges between 4.02% and 7.11% (Z. M. Younossi et al. 2023), both depending on the country. MASH—previously NASH—is a stage characterized by hepatocellular ballooning and lobular inflammation. These liver changes can arise from various causes, with an unhealthy diet, particularly excessive fat intake, being a key factor in its etiology (Polyzos and Mantzoros 2016; Stefan, Kantartzis, and Häring 2008).

Physiologically, MASLD is characterized by the presence of steatosis, that is, lipid accumulation in more than 5% of hepatocytes (Cobbina and Akhlaghi 2017). If this stage progresses to MASH, we may also observe fibrosis, inflammation, hepatocellular injury, and hepatocellular ballooning, which is a type of apoptosis characterized by an increase in cell size (Paternostro and Trauner 2022). Besides, cirrhosis develops as a result of inflammation and fibrosis caused by MASH (Ginès et al. 2021), and it can increase the risk of developing carcinoma, which is the most common type of liver cancer (Hartke, Johnson, and Ghabril 2017).

Furthermore, MASH disease can be associated with an affectation of the nervous system through several potential pathways, including gene expression, hormone and neurotransmitter secretion, immune function, neuronal activity, and remodeling of synaptic dendritic spines (Albillos, de Gottardi, and Rescigno 2020).

Chronic stress is a risk factor commonly related to MASH (Stefanaki et al. 2023). It causes activation of the hypothalamic‐pituitary‐adrenal axis, which raises cortisol levels and triggers pro‐inflammatory processes, influencing the progression of liver disease (Shea et al. 2021; Lupien et al. 2018; Snyder et al. 2011). Alterations in areas such as the hippocampus and prefrontal cortex are associated with deficits in cognitive performance, including inhibition, attention, learning, decision‐making, and memory (Price and Duman 2020).

In addition, the vagus nerve transmits signals to the brain, and its afferents can be stimulated by intestinal microbes (Cheon and Song 2022; M. Yan et al. 2023). The gut mucosal barrier within the intestinal epithelium protects against bacteria and toxins; it also connects the gut to the liver (Albillos, de Gottardi, and Rescigno 2020; Cheon and Song 2022). Damage to this barrier increases permeability, allowing pathogens to enter the liver, affecting bile acid metabolism (Cheon and Song 2022; Milosevic et al. 2019; Tilg, Adolph, and Trauner 2022), which results in gut dysmotility, dysbiosis, inflammation, liver fibrogenesis, systemic inflammation, and neuroinflammation, ultimately causing cognitive dysfunction (Albillos, de Gottardi, and Rescigno 2020; Cheon and Song 2022; Milosevic et al. 2019; Tilg, Adolph, and Trauner 2022). Dysbiosis produces ammonia, which alters brain pH, membrane potential, cell metabolism, and neurotransmission, and causes astrocyte swelling and brain edema (Mancini et al. 2018). Dysbiosis also leads to vagal remodeling (Berding et al. 2021). Moreover, our group found that dysbiosis and gut‐derived microbiota metabolites caused by MASH, along with ammonia, generate neurotoxic injury, reflected in metabolic and functional brain regional deficits (Higarza et al. 2019).

An important mechanism involved in the gut–liver–brain relationship is neuroinflammation (Berding et al. 2021), which is likely to occur on account of an elevated concentration of inflammatory cytokines, released as a consequence of systemic inflammation, which severely impairs the blood–brain barrier (Kelty et al. 2023). This barrier separates the central nervous system (CNS) from the peripheral circulatory system and protects the brain from pathogen entry. Chronic inflammation increases its permeability, allowing inflammatory cells and molecules to enter the brain (Kwon and Koh 2020; Mukherjee et al. 2023).

There is a link between neuroinflammation and the presence of reactive glial cells, specifically microglia and astrocytes (Kelty et al. 2023; Mukherjee et al. 2023). Astrocytes, which are more abundant, are involved in the regulation of various neuronal processes and homeostasis, in addition to the immune response (Kelty et al. 2023; Kwon and Koh 2020). However, the role of astrocytes in the neurobiology of MASH has not been fully researched.

Although there is some evidence of the effect of MASH on cognition (Higarza et al. 2021, 2019), this subject has been poorly addressed. Some studies in humans with MASLD have pointed out a certain cognitive decline through the evaluation of different tests such as the Montreal Cognitive Assessment (Celikbilek, Celikbilek, and Bozkurt 2018; Filipović et al. 2018) and the Frontal Assessment Battery (Moretti, Caruso, and Gazzin 2019). Moreover, Seo et al. (2016) described that these patients showed an impaired execution of the serial digit learning test, which involves learning, recall, and concentration, whereas the simple reaction time test and symbol‐digit substitution test were performed correctly, which could indicate conserved visual‐motor speed and visual attention. Thereby, most of the research in this field has been addressed to a generic cognitive outcome, which does not allow for elucidating the specific implications of the disease. Some studies conducted by Higarza et al. (2021, 2019) had particularly shown a relation between MASH and short‐term memory impairment, social recognition, novel object recognition, and spatial working memory deficits. These deficits are associated with alterations in the metabolic activity of certain brain regions, including the prefrontal cortex and the hippocampus (Higarza et al. 2021). In this way, the potential affectation of memory has been focused on explicit memory, and some other processes such as attention and associative learning have been overlooked.

Thus, the aim of the research is to conduct a psychobiological exploration of MASH in adult male rats, which were induced to eat a high‐fat and high‐cholesterol diet for 14 weeks. We assessed the acoustic startle response and alertness through the prepulse inhibition (PPI) paradigm and the associative learning by the use of the passive avoidance test. Also, we explored the astrocyte density in the prefrontal cortex and hippocampus.

## 2 Materials and Methods

### 2.1 Subjects

**FIGURE 1:**
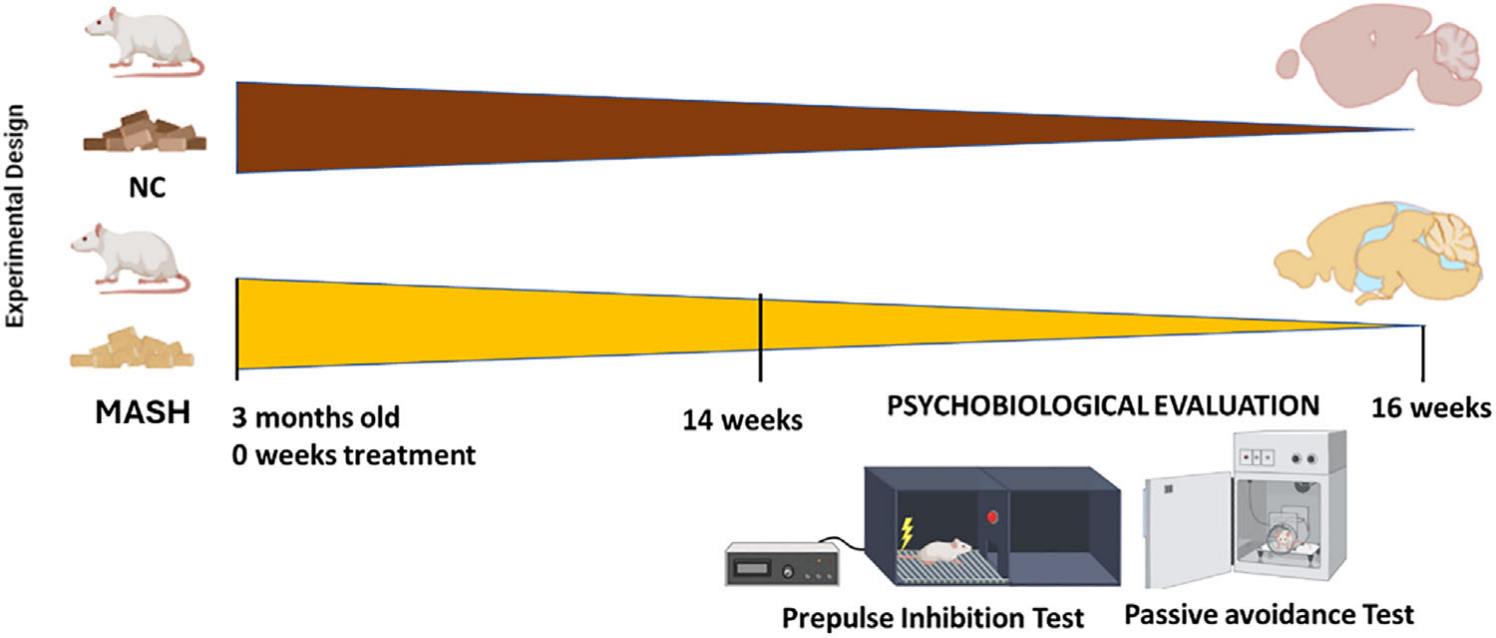
Experimental design. Timeline of the experiment. Each group was administrated their respective diets for 14 weeks. Two experimental groups (*n* = 10 per experimental group) of male Sprague–Dawley rats were used. The NC group was dispensed a normal chow, the MASH group was fed with a high‐fat, high‐cholesterol diet. The behavioral evaluation started on Weeks 14–16 at the end of which the animals were sacrificed and the samples were collected.

A total number of 20 male Sprague‐Dawley rats were used (200–220 g at the start of the experiment). The rats were randomly divided into two groups: the normal control (NC) group (*n* = 10) and the MASH group (*n* = 10). The control group was administered a diet made of 13% kcal fat, 20% kcal proteins, and 67% kcal carbohydrates (Envigo‐2914), whereas the MASH group was fed with a 65% kcal fat, 20% kcal proteins, and 15% kcal carbohydrates diet (Research Diets, New Brunswick, NJ, USA; #D09052204). Both groups received their respective diets for 14 weeks, in accordance with our previous study that demonstrates that this animal model displays the pathophysiological features of the disease (Higarza et al. 2019; Figure 1). Specifically, in this prior article, we described that this model exhibited disease criteria at physiological, histological, and biochemical levels. Among the most significant alterations, the MASH group displayed increased portal pressure, elevated levels of alanine aminotransferase (ALT), aspartate aminotransferase (AST), and glucose, which represent a characteristic pattern of liver dysfunction. In addition, they exhibited altered cholesterol profiles and hyperammonemia.

In order to monitor the effects of the diet, the animals were systematically weighed weekly. After 14 weeks on the diet, the animals were subjected to the behavioral tasks.

All the animals had ad libitum access to tap water and were maintained at constant room temperature (22°C ± 2°C), with a relative humidity of 65% ± 5% and an artificial light‐dark cycle of 12 h (8 am–8 pm/8 pm–8 am). Behavioral testing was conducted between 8 am and 1 pm. The procedures and manipulation of the animals were realized according to the European Union Directive and European Union Council 2010/63/UE. The National Legislation, in accordance with this Directive, is defined in Royal Decree No. 53/2013 on the use of animals in research. The study was approved by the ethics committee of the University of Oviedo (PROAE 25/2018).

### 2.2 Acoustic Startle Response and PPI Test

Startle response and alertness were studied through the PPI of the acoustic startle paradigm. This test was carried out in an SR‐LAB apparatus (Cibertec S.A., Madrid, Spain) composed of a plexiglass box (28 × 15 cm), in which each animal was individually placed, and that rested on a piezoelectric transducer that detected the vibrations derived from the movement of the animal. The protocol, similar to Banqueri, Méndez, and Arias (2017) and Valsamis and Schmid (2011), started with a 5 min acclimation period of 65 dB background noise and was followed by two sessions. In the first one, the animal was exposed to 30 pulse trials of 115 dB and 20 ms, which were separated in time by random intervals of 10 or 30 s. The second session consisted of 50 trials randomly applied; in 10 of them, the 115 dB—20 ms pulses were presented alone, and, in the rest, they were preceded by a 20 ms prepulse of 75 or 85 dB appearing 30 or 100 ms before the pulse, which resulted in 10 trials with each combination. The percentage of PPI was calculated as [(mean startle amplitude in first session trials − mean startle amplitude in each condition of second session trials)/mean startle amplitude in first session trials] × 100.

### 2.3 Passive Avoidance Test

Associative learning was assessed through a passive avoidance test in a shuttle box composed of two plexiglass chambers (23 × 22 × 22 cm each) connected by a sliding door (37.5 × 7 cm) (7552 ‐ Ugo Basile, Milan, Italy). One of the chambers was lit (24 V 5 W), whereas the other one was dark. The grid floor of the apparatus consisted of 0.3 cm diameter stainless bars, separated by 1 cm, which were connected to a shock scrambler (7551 ‐ Ugo Basile, Milan, Italy) that controlled the duration of the trial, the door‐opening delay, the latency to crossing to the dark chamber, and the duration and intensity of the shock.

The protocol, similar to Banqueri, Méndez, and Arias (2017) and Arias, Méndez, and Arias (2015), consisted of four phases. In all of them, the animal was placed in the lit chamber, and after 10 s, the sliding door was opened. When the animal introduced its complete body in the dark chamber, the crossing latency was recorded; after 3 s, the door was closed, and 10 s later, the subject was removed from the apparatus. The maximum duration of each trial was 300 s.

In the first phase, the animal was habituated to the apparatus, and 1 h later, the acquisition phase was carried out, in which, when the subject crossed to the dark chamber, an electric shock of 0.9 mA for 3 s was delivered. The retrieval was assessed 1 day and 1 week after the acquisition trial, proceeding with the same protocol with no electric shock. Latencies to crossing to the dark chamber were recorded in each trial.

### 2.4 Brain Tissue Collection

For the astrocyte density study, a total of 11 subjects were utilized, with 5 in the MASH group and 6 in the control group. Once the behavioral experiments were finished, the animals were decapitated. Their brains were then fixed in a 4% formaldehyde solution (Fisher Scientific, Spain) for 48 h, dehydrated in a graded ethanol series (70°–100°) (VWR, Spain), subjected to two butyl acetate immersions (J.T. Baker, USA), and subsequently embedded in paraffin for histological examination. Each subject had 8 sections cut at a 30 µm thickness: 4 from the prefrontal cortex and 4 from the hippocampal region. The regions of interest were anatomically defined according to the atlas of Paxinos and Watson (2007). Glial fibrillary acidic protein (GFAP) immunocytochemistry was conducted on regions within the bregma coordinates of 3.20–2.70 mm for the cingulate cortex (CG), prelimbic cortex (PrL), and infralimbic cortex (IL), and −3.30 to −3.60 mm for the CA1, CA3, and dentate gyrus (DG) subfields of the dorsal hippocampus.

### 2.5 GFAP Immunocytochemistry

The protocol for GFAP immunocytochemistry was applied similarly to Zorzo et al. (2019). In the first step, sections were immersed in xylene‐paraffin, then hydrated by immersing them in a decreasing chain of alcohol (100%‐96%‐80%) and distilled water. Sections are then washed in TBS‐Triton and incubated in bovine‐blocking serum (Sigma) diluted 1% in TBS. Next, washes are repeated, and incubation in rabbit polyclonal anti‐GFAP primary antibody (1:800) (Dako) diluted in bovine serum (1% in TBS) is started. The control slide is incubated in TBS, without diluted antibody. Then, sections are washed with TBS‐Triton, incubated with biotinylated goat anti‐rabbit IgG (1:400) secondary antibody (Pierce) diluted in bovine serum (1% in TBS), washed again with TBS‐Triton, and incubated with avidin‐biotin‐peroxidase complex (Vectastain). Next, washed with TBS and with TBS‐Triton. Then visualized with diaminobenzidine tetrahydrochloride (DAB), rinsed with distilled water, and dehydrated by immersing them in an increasing chain of alcohol (80%‐96%‐100%). Next, immersed in xylene‐alcohol. Finally, the slides are mounted with Entellan mounting fluid and glass coverslips.

### 2.6 GFAP Quantification

A microscope (Leica, Germany) and the Leica Application Suite X software (Leica Microsystems, Germany) were used to quantify the number of astrocytes. The prefrontal cortex areas were observed by using a 20× objective, while for the hippocampal regions, a 10× objective was employed. Dissectors were placed by random sampling. In the prefrontal cortex regions, one dissector (150 × 150 µm^2^) was placed in each of the four sections of the CG, PrL, and IL for each subject. In the hippocampal region, two dissectors (100 × 100 µm^2^) were placed in each of the four sections of the CA1, CA3, and DG subfields for each subject.

### 2.7 Statistical Analysis

All data were analyzed using SigmaStat 4.0 software (Systat, San Jose, CA, USA) and expressed as means ± SEM. Astrocyte density, startle response, and percentage of PPI in each condition, as well as latencies to cross to the dark chamber in the passive avoidance task, were compared between groups through Student's *t*‐test for independent samples. Mann–Whitney *U* test for independent samples was used when normality or equal variances across groups failed. Final outcomes were considered significant when *p* < 0.05.

## 3 Results

### 3.1 Acoustic Startle Response and PPI

**FIGURE 2:**
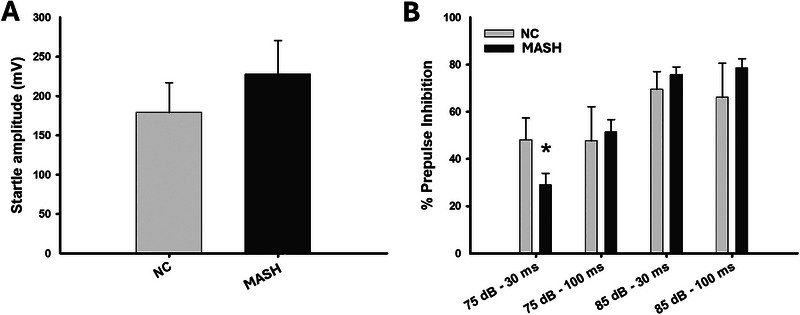
Prepulse inhibition task. (A) Bar charts (mean ± SEM) represent startle amplitude. (B) Bar charts (mean ± SEM) represent prepulse inhibition in each condition. MASH group displayed less inhibition than NC group in the 75 dB prepulse condition presented 30 ms before the pulse (*p *< 0.05).

The PPI paradigm was used to assess startle response and alertness. The startle response was statistically similar in both groups (*U* = 65.000, *n*_1_ = 10, *n*_2_ = 10, *p* = 0.273) (Figure 2A); however, the MASH group displayed a significantly reduced percentage of inhibition to the 75 dB prepulse applied 30 ms before the pulse (*U* = 20.000, *n*_1_ = 10, *n*_2_ = 10, *p* = 0.026). Differences between groups in the rest of the conditions were not observed (75 dB—100 ms: *U* = 35.000, *n*_1_ = 10, *n*_2_ = 10, *p* = 0.273; 85 dB—30 ms: *U* = 57.000, *n*_1_ = 10, *n*_2_ = 10, *p* = 0.623; 85 dB—100 ms: *U* = 55.000, *n*_1_ = 10, *n*_2_ = 10, *p* = 0.734) (Figure 2B). The MASH group exhibited impaired inhibition to the less intense pulse that was presented closest in time to the main pulse, indicating a mild impairment of alertness.

### 3.2 Passive Avoidance

**FIGURE 3:**
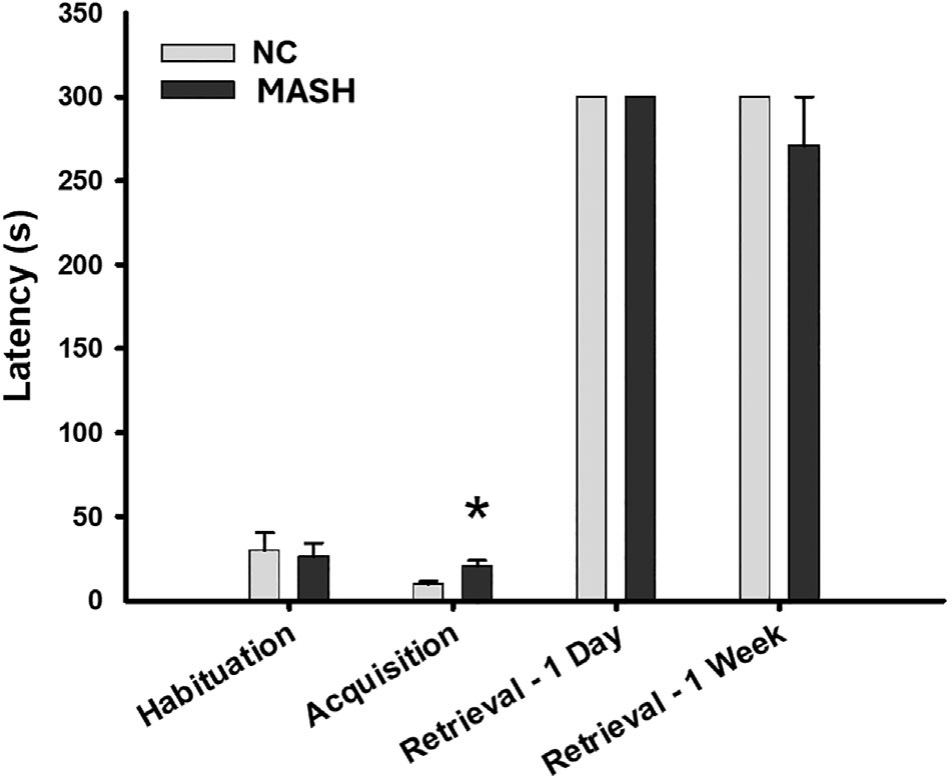
Passive avoidance task. Bar charts (mean ± SEM) represent latencies to crossing to the dark chamber during habituation, acquisition, retrieval after 1 day and 1 week. MASH group displayed higher latency in the acquisition trial than the NC group (* *p *< 0.05) which tends to decrease over time.

The passive avoidance paradigm was used to study associative learning. In the habituation trial, there were no significant differences between groups in the latencies to crossing to the dark chamber (*U* = 46.500, *n*_1_ = 10, *n*_2_ = 10, *p* = 0.791); however, in the acquisition trial, the MASH group displayed a significantly higher latency than the NC group (*t*_17_ = 2.761, *p* = 0.0129). Crossing latencies after the administration of the electric shock did not differ statistically between groups, nor after one day (*U* = 50.000, *n*_1_ = 10, *n*_2_ = 10, *p* = 1.000), nor when 1 week had elapsed (*U* = 45.000, *n*_1_ = 10, *n*_2_ = 10, *p* = 0.317), since most of the subjects stayed in the light chamber (Figure 3). These results indicate a lack of exploratory behavior in the MASH group leading to a delay in the acquisition regarding the formation of a contextual representation associated with a shock.

### 3.3 Astrocyte Density

**FIGURE 4:**
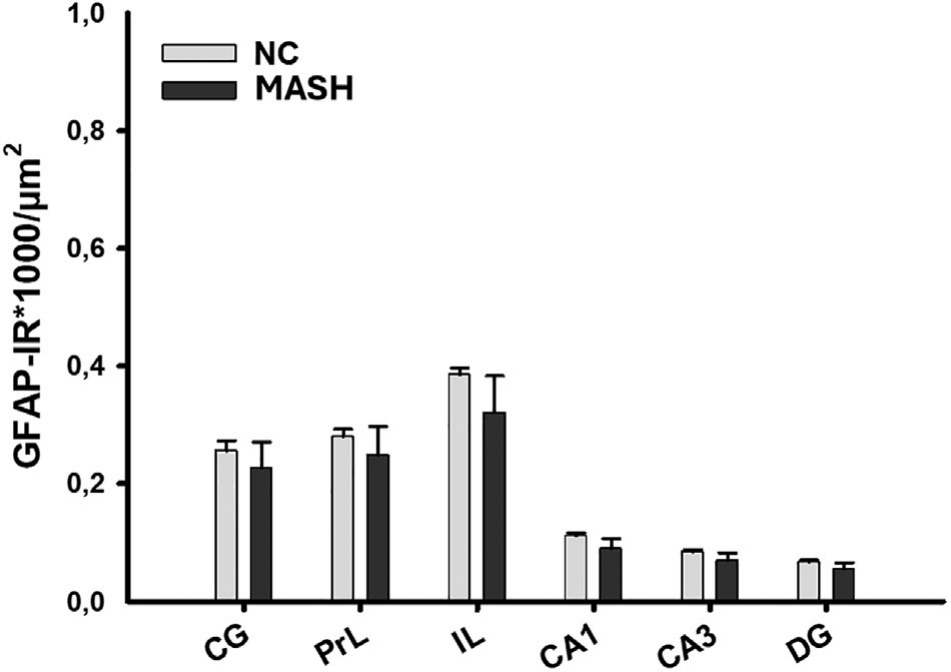
Astrocyte density (GFAP immunoreactive cells (IR)*1000/ area). Bar charts (mean ± SEM) represent the number of GFAP‐IR in each brain area. CG: cingulate cortex; PrL: prelimbic cortex; IL: infralimbic cortex; CA: cornu ammonis; DG: dentate gyrus.

Regarding the results of astrocyte density, no differences were found in any of the regions studied (*p* > 0.05), such as CG (*t*_9_ = 0.570, *p* = 0.583); PrL (*t*_9_ = 0.887, *p* = 0.398); IL (*t*_9_ = 0.167, *p* = 0.871); CA1 (*t*_9_ = 0.855, *p* = 0.415); CA3 (*t*_9_ = 0.224, *p* = 0.828); DG (*t*_9_ = 0.222, *p* = 0.829) (Figure 4).

## 4 Discussion

Individuals with MASH have been reported to experience cognitive impairment (Kjærgaard et al. 2021), with the etiology and specific cerebral dysfunction yet to be thoroughly characterized. In our study, we used a high‐fat, high‐cholesterol diet model, which emulates MASH and other typical features of the disease (Higarza et al. 2019). Previously, we also reported the impact of this MASH‐like condition on metabolic and functional processes. Brain tissue demonstrated lower levels of metabolic brain activity in the prefrontal cortex and hippocampus among other brain regions, accompanied by a decrease in dopamine levels in the prefrontal cortex. In addition, metabolic alterations in other obesity and MASLD combined models were accompanied by microglial and astrocytic morphological changes. However, less is known about the independent impact of MASH on brain astrogliosis in prefrontal and hippocampal brain regions and on the complete behavioral affection.

PPI is a neuropsychological process during which a weak sensory stimulus (“prepulse”) attenuates the motor response (“startle reaction”) to a subsequent strong startling stimulus. It is measured as a surrogate marker of sensorimotor gating in patients suffering from neuropsychological diseases, as well as in corresponding animal models. A variety of studies has shown that PPI of the acoustical startle reaction comprises three brain circuitries for (i) startle mediation, (ii) PPI mediation, and (iii) modulation of PPI mediation. Several cortico‐limbic areas modulate PPI mediation, like the septohippocampal system and medial prefrontal cortex, among others (for review, see Swerdlow, Geyer, and Braff 2001; Koch and Fendt 2003). During the PPI paradigm, where no problem‐solving, decision‐making, or other cognitive/executive actions are needed, those brain regions may be recruited for PPI modulation. It is therefore thought that the PPI modulation network controls sensorimotor gating by influencing the activity of the PPI mediation network continuously (Rohleder et al. 2014), related to attentional and emotional states (Li et al. 2009). Blumenthal, Reynolds, and Spence (2015), with the “protection hypothesis,” along with data from Rohleder et al. (2016), suggested that cortico‐limbic areas are involved in protecting the processing of the prepulse. In this model, due to damage in the prefrontal cortex and hippocampus, MASH animals displayed a conserved startle response and a reduced PPI with the 75 dB—30 ms prepulse, the weaker and closer‐to‐the‐stimulus prepulse, indicating an alteration in alertness.

Regarding the status of attention processes in this disease, different results were found in the literature. Some studies described a worsened execution in sustained attention tasks in humans (Filipović et al. 2018), whereas a correct performance has also been found in this type of task (Celikbilek, Celikbilek, and Bozkurt 2018). Previous studies using an administration of high‐fat diet in animal models had shown an altered PPI as a consequence of dopaminergic dysfunction (Labouesse et al. 2013; Wakabayashi et al. 2015; Wakabayashi and Kunugi 2018). In our previous study addressing the neurobehavioral status of this MASH model, we hypothesized that the behavioral changes and neuronal deficits could be derived from the dopaminergic dysregulation caused by the observed hyperammonemia and decreased production of short‐chain fatty acids in this network (Higarza et al. 2019). So, the alteration caused by the dopaminergic dysregulation in some of the PPI‐mediation brain regions could be underlying the behavioral results observed in the PPI test.

Passive avoidance conditioning is one of the simplest and most rapid behavioral paradigms for studying learned aspects of associative learning and defensive behavior. Rats have an innate preference for dark environments; however, upon entering the dark compartment, they receive an electric shock. Under this paradigm, when the subject is later returned to the same chamber in which the conditioning occurred, but in the absence of the aversive stimulus (i.e., the next day), it exhibits the opposite response compared to the previous day (i.e., it does not cross into the aversive chamber). This aversive stimulus conditions them to remain in the light chamber. Our results indicated that the MASH animal group showed increased latencies to cross into the dark chamber compared to the control group; thus, they exhibited reduced exploratory behavior, which could be supporting previous results from our group (Higarza et al. 2021), where novel object recognition was altered in this animal model. These results are opposite to Ganji et al. (2017) and Rezvani‐Kamran et al. (2017). These studies developed a rat model administered with a high‐fat diet for 3 months; however, the characteristics of these diets must also be taken into account. The first study used a 45% energy‐from‐fat diet, but it did not specify the source of the fat. On the other hand, the second study employed a 67.7% energy‐from‐fat diet, which may reflect an acute impact on brain damage compared to our model, which involved a 4‐month diet consisting of 45% kcal fat derived from cocoa butter.

Animal studies have demonstrated that high‐calorie diets detrimentally affect the structure and function of the hippocampus, a critical brain region for learning and memory (Molteni et al. 2004; Farr et al. 2008; Stranahan et al. 2008; Karimi et al. 2013). Saturated fat, hydrogenated fat, and cholesterol notably impair memory and hippocampal morphology (Granholm et al. 2008). In addition, high‐fat diets are linked to elevated levels of cholesterol and triglycerides in the serum and liver, increased cholesterol accumulation in the hippocampus, and lipid peroxidation, which contribute to hippocampal alterations (Stranahan et al. 2011). Male rats on high‐fat diets have been found to exhibit reduced hippocampal neurogenesis (Lindqvist et al. 2006). Diets high in saturated fats and simple sugars also compromise the expression of several neurotrophic factors that support and enhance hippocampal plasticity (Molteni et al. 2002; Stranahan et al. 2008). Furthermore, existing literature has demonstrated alterations in the recognition of novel objects and spatial working memory as a result of high‐fat, high‐cholesterol diets (Higarza et al. 2021, 2019). In humans, several studies have reported that patients with steatotic liver disease exhibit specific cognitive impairments, including deficits in visuospatial processing and frontal‐associated functions (Celikbilek, Celikbilek, and Bozkurt 2018; Moretti, Caruso, and Gazzin 2019).

Moreover, steatosis triggers inflammatory and metabolic events that can affect the function of astrocytes, turning them into a reactive and detrimental state as it increases levels of inflammation, steatosis, fibrosis, and metastasis and promotes the progression of MASLD to more severe stages (Komaniecki et al. 2023; J. Yan et al. 2022). In these severe stages, a higher degree of neuronal loss, neuroinflammation, and astrocytes is observed (Balzano et al. 2018; Jaeger, DeMorrow, and McMillin 2019). Astrocytes respond to the inflammation caused by cytokines, as they play an important role in regulating homeostasis and neuronal processes and are also responsible for the immune response (Kelty et al. 2023; Kwon and Koh 2020). Despite the potential importance of astrocytes in relation to MASH, there is little literature regarding this subject. In this regard, our results showed that astrocyte density remained similar in both experimental groups, despite the development of MASH. Considering that increased GFAP protein expression is a commonly used molecular marker for the study of reactive astrocytes, this implies that we did not find reactive astrogliosis in the MASH model.

The few investigations conducted on this topic found that reactive cortical astrogliosis develops in an obese MASH model (Hadjihambi et al. 2023), suggesting that obesity produces specific changes in the gut microbiota through the gut–brain axis, which alters the levels of pro‐inflammatory cytokines that cause systemic inflammation and neuroinflammation (Minaya et al. 2020). In a recent study involving rats fed a high‐fat diet, where 43% of the kcal from fat was derived from soybean oil and lard, over a period of 25 weeks, it was observed that the high‐fat diet induced peripheral insulin resistance and elevated both systemic and neuroinflammation, as evidenced by increased levels of tumor necrotic factor in the blood, liver, and brain (Bittencourt et al. 2022). Neuroinflammation was further characterized by enhanced astrogliosis in the substantia nigra and ventral tegmental area, with the latter also exhibiting oxidative stress, indicated by elevated nitrotyrosine levels (Bittencourt et al. 2022). Those differences in results could be explained by the use of palmitate in the given diet, which constitutes palmitic acid, the predominant saturated fatty acid in the human body and a constituent of soybean oil commonly included in high‐fat diet pellets utilized in obesity animal models (Walsh 2007). However, in our study, palmitic acid constitutes approximately 19% of the total 45% of the kcal from fat, primarily derived from cocoa butter. This avoids the direct effects of palmitic acid that have been described in the context of obesity, which do not apply to MASH. In this regard, both experimental groups did not exhibit differences in weight. To our knowledge, this is the first study to investigate astrocyte function in a nonobese MASH model, and little is known about the relationship between MASH and astrocytes in the absence of weight gain. Our findings indicate that a nonobese MASH animal model does not initiate the same inflammatory processes as those observed with obesity.

## 5 Conclusion

MASLD spectrum is a growing health problem worldwide. This work expands the knowledge about this disease and its relationship with cognitive impairment and glial cell activity. Our results indicate that MASH, derived from the administration of a high‐fat and high‐cholesterol diet, was associated with mild alertness dysfunction and differences in the associative learning. Furthermore, MASH was not observed to be linked with an astrocyte density increase in the prefrontal cortex nor in the hippocampus. We highlighted the importance of distinguishing between obesity and MASH, as well as closely examining the origin of the fat components to ensure that our results can be effectively translated into clinical practice. Overall, these findings emphasize the necessity for further investigation into the relationship between attention processes, associative learning, neuroinflammation, and dopaminergic function in the context of MASH, as they may have significant implications for understanding the cognitive deficits associated with this condition.

## Author Contributions

**Sara G. Higarza**: methodology; software; data curation; investigation; validation; formal analysis; visualization; writing–original draft. **Marina De Antón‐Cosío**: investigation; data curation; formal analysis; writing–original draft. **Candela Zorzo**: supervision; validation; visualization; formal analysis; writing–review and editing. **Jorge L. Arias**: conceptualization; funding acquisition; methodology; project administration; resources; supervision; writing–review and editing. **Natalia Arias**: conceptualization; funding acquisition; methodology; project administration; supervision; writing–original draft; writing–review and editing.

## Ethics Statement

The study was approved by the ethics committee of the University of Oviedo (PROAE 25/2018).

## Conflicts of Interest

The authors declare no conflicts of interest.

### Peer Review

The peer review history for this article is available at https://publons.com/publon/10.1002/brb3.70222.

## Data Availability

The data that support the findings of this study are available from the corresponding author upon reasonable request.
